# Machine learning modeling for the prediction of plastic properties in metallic glasses

**DOI:** 10.1038/s41598-023-27644-x

**Published:** 2023-01-07

**Authors:** Nicolás Amigo, Simón Palominos, Felipe J. Valencia

**Affiliations:** 1 Facultad de Ingeniería, Arquitectura y Diseño, Universidad San Sebastián, Bellavista 7, 8420524 Santiago, Chile; 2 Facultad de Ciencias, Ingeniería y Tecnología, Escuela de Ingeniería Industrial, Universidad Mayor, Santiago, Chile; 3 Departamento de Computación e Industrias, Facultad de Ciencias de la Ingeniería, Universidad Católica del Maule, 3480112 Talca, Chile; 4 Centro para el Desarrollo de la Nanociencia y la Nanotecnología, CEDENNA, Avda. Ecuador 3493, 9170124 Santiago, Chile

**Keywords:** Materials science, Nanoscale materials, Structural materials

## Abstract

Metallic glasses are one of the most interesting mechanical materials studied in the last years, but as amorphous solids, they differ strongly from their crystalline counterparts. This matter can be addressed with the development and application of predictive techniques capable to describe the plastic regime. Here, machine learning models were employed for the prediction of plastic properties in CuZr metallic glasses. To this aim, 100 different samples were subjected to tensile tests by means of molecular dynamics simulations. A total of 17 materials properties were calculated and explored using statistical analysis. Strong correlations were found for stoichiometry, temperature, structural, and elastic properties with plastic properties. Three regression models were employed for the prediction of six plastic properties. Linear and Ridge regressions delivered the better prediction capability, with coefficients of determination above $\sim$80% for three plastic properties, whereas Lasso regression rendered lower performance, with coefficients of determination above $\sim$60% for two plastic properties. Overall, our work shows that molecular dynamics simulations together with machine learning models can provide a framework for the prediction of plastic behavior of complex materials.

## Introduction

Metallic glasses (MGs) are one of the most interesting materials synthesized in the last years due to their exceptional combination of strength and elasticity ^1,2^. As any other amorphous material, MGs are strongly sensitive to several parameters such as atomic composition, ^3,4^ synthesis process, ^5^ cooling rate ^6^, or processing requirements ^7^. A relevant difference with their crystalline counterparts is that MGs possess limited ductility, making difficult the investigation of plasticity. This way, computational simulations and predictive models can shed light on the characterization of mechanical response, as well as to establish relationships between materials properties.

Supervised learning algorithms have been applied in materials science for different research purposes at both experimental and theoretical levels. Sun et al.^8^ predicted the efficiency of organic photovoltaic materials by using different types of ML models on a database of over 1700 donor materials. Design of molecular structures with tailored chemical and structural properties was shown by Gebauer et al.^9^, where generative neural networks were used to this aim. Drug discovery is another research area that has benefited from ML, as shown in the review of Vamathevan and co-workers, where applications of several techniques are discussed ^10^. Other authors have contributed in the acceleration of first principles calculations ^11,12^, which are accurate in materials modeling but lack of computational efficiency to handle large scale systems. On the other hand, machine learning stands for an alternative approach to construct classical inter–atomic potentials with adaptive force fields in function of the atomic environment leading to more reliable energy calculations within their boundary of validity ^13^. ML techniques have also been employed to predict macroscopic materials properties. For example, melting points of ionic liquids were predicted using tree–based ensemble methods on a large combination of cations and ions ^14^. Gaussian process regression was carried out to design copper alloys with enhanced strength and electrical conductivity ^15^. Regarding mechanical behavior, neural networks have been used to extract elastoplastic properties of engineering alloys ^16^, yield strength and ultimate tensile strength of aluminum alloys ^17^, stiffness, strength, and toughness of composites ^18^, yield stress of high entropy alloys ^19^, and both Young’s modulus and ultimate tensile strength of graphene–reinforced metal matrix nanocomposites ^20^.

Metallic glasses (MGs) are materials with remarkable properties, but suffer from lack of ductility ^21,22^. Extensive research has been conducted at the atomic scale to address this matter, mainly based on molecular dynamics simulations (MD). Some examples include cooling history and its influence on the initial plastic flow ^23^, shear localization during homogeneous deformation ^24^, atomic composition and shear band (SB) formation ^25^, shear transformation zones (STZs) and SB structures ^26,27^, evolution of atomic structure during plastic regime ^28,29^, among many others ^30^. Despite the vast amount of literature, no systematic relationships have been established between structural, elastic and plastic properties. In this work, we propose ML models to predict the plastic behavior of CuZr MGs based on several materials properties set as predictors (input features), including stoichiometry, sample dimensions, structural properties, temperature, potential energy, and elastic properties. To this aim, 100 different samples were simulated using MD simulations and subjected to tensile tests to obtain plastic quantities such as yield stress, flow stress, ultimate tensile stress, among others. Statistical analysis and ML techniques were carried out to explore correlations between properties and to establish predictive models.

## Methods

### Simulation setup

A set of 100 $\hbox {Cu}_{x}$ $\hbox {Zr}_{100-x}$ MGs were constructed under different random conditions. Alloy composition *x* ranged from 36 to 64 atomic percent, while the initial dimensions of $L_x$, $L_y$, $L_z$ were in the range of 16.2–25.9 nm, 6.48–9.72 nm, and 1.62–4.86 nm, respectively. The larger length of $L_x$ was deliberately imposed in order to obtain a larger axial dimension to conduct tensile tests. Periodic boundary conditions were set in all directions. Samples were kept in the melt at 2500 K for 10 ns and then quenched to 50 K at a constant cooling rate $R_c$, which was set in the range of $10^{10}--10^{11}$ K/s. Lower values were excluded to avoid long simulation times. Relaxation at a target temperature (*T*) was conducted for 1.0 ns while keeping zero pressure, where *T* ranged from 50 to 300 K. Thus, atomic composition, dimensions, cooling rate, and temperature were different for the 100 samples. MGs were modeled with the interatomic potential developed by Cheng et al. ^31^. This interatomic potential was fitted according to a wide variety of crystal structures simulated following ab initio MD, together with elastic constants and phonon frequencies obtained from experiments. Validation of the model was performed against experimental and ab initio data, including cohesive energies, lattice parameters, bulk modulus, among others. Furthermore, the interatomic potential has been employed in several works, such as dynamic arrest ^32^ and shear modulus prediction ^33^. Uniaxial tensile tests were carried out with a constant strain rate of $10^8~\hbox {s}^{-1}$ by loading the *x* direction and keeping zero pressure along the *y* and *z* directions. Temperature was kept constant at *T*, which corresponds to the relaxation temperature of the sample. MD simulations were performed with the LAMMPS package ^34^ using an integration timestep of 1.0 fs.

### Machine learning modeling

**Figure 1:**
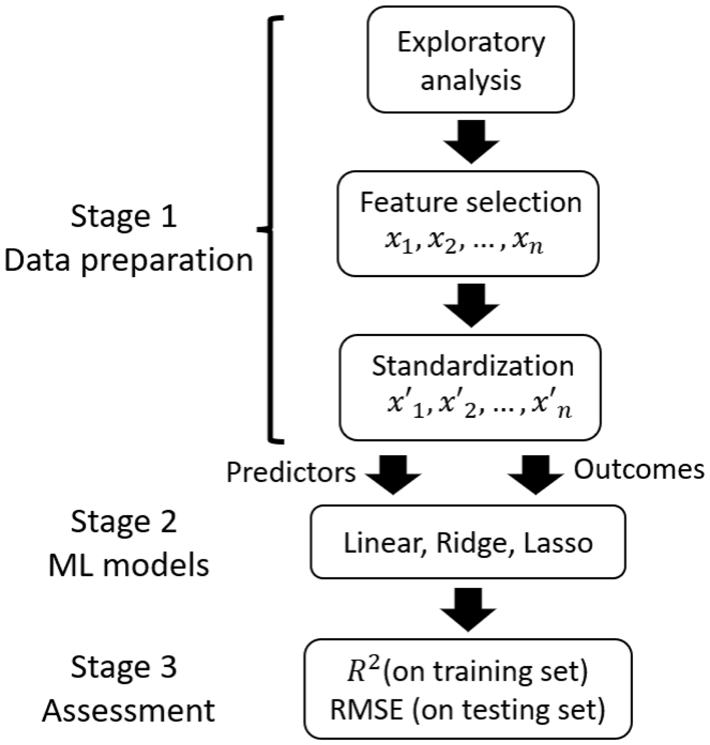
Scheme of the processing pipeline: from data inspection to model assessment. Structural and elastic properties were employed as predictors, while plastic properties were considered as responses.

ML modeling involves several steps in order to be conducted. Data acquisition and inspection are required for proper feature selection, followed by feature engineering for supervised learning. Then, statistical metrics are computed to assess the performance of the ML models. A brief summary of the processing pipeline is displayed in Fig. 1.

In stage 1, a total of 17 materials properties, called features hereafter, were retrieved from the 100 samples. Thus, each feature had a total of 100 observations. A brief description of each feature is given in the following. Percentages of Cu species together with the total number of atoms $N_a$, were obtained from the total atomic composition. The percentage of Zr atoms was not considered since it depends directly on Cu. Dimensions ($L_x, L_y, L_z$) together with temperature (*T*) and cooling rate ($R_c$) were also considered. The coordination number (*CN*) and the average degree of five–fold local symmetry (*W*) were obtained from structural characterization, while the Young’s Modulus (*E*), Poisson’s Ratio ($\nu$), resilience ($u_R$), yield stress ($\sigma _Y$), ultimate tensile stress ($\sigma _{UTS}$), flow stress ($\sigma _F$), drop stress ($\sigma _D$), and toughness ($u_T$) were obtained from the stress–strain curves. Here, *E* and $\nu$ correspond to elastic properties, while $u_R$, $\sigma _Y$, $\sigma _{UTS}$, $\sigma _F$, $\sigma _D$, and $u_T$ correspond to plastic properties. We note that resilience and yield stress represent the beginning of plasticity, and thus can be considered as plastic indicators ^35^. A similar approach was adopted by Zhang et al. ^19^. In this work, $\sigma _Y$ was calculated following the offset criterion with $\varepsilon _{offset} = 0.002$, $\sigma _{UTS}$ corresponded to the maximum stress, $\sigma _F$ was obtained from the average stress between 0.15–0.20 strain, and $\sigma _{D}$ was the difference $\sigma _{UTS}-\sigma _{F}$. More details on the calculation of the 17 features can be found in the supplementary material.

Statistical exploration was performed by computing the mean, the standard deviation, quartiles, among others. The Spearman’s correlation coefficient $\rho$ was calculated to assess the degree of correlation between each pair of features. The coefficient was obtained as

1 $$\begin{aligned} \rho = 1 - \frac{6\sum _{i=1}^n d_i^2}{n(n^2-1)}, \end{aligned}$$

where $d_i$ is the difference between the two ranks of the pair of features, and *n* is the number of observations. Features with relevant degrees of correlation were retained in the pipeline and standardization was performed on them as follows

2 $$\begin{aligned} x_{stand} = \frac{x - \overline{x}}{s_x}, \end{aligned}$$

where $\overline{x}$ and $s_x$ are the mean and the standard deviation of the sample *x*, respectively. Such transformation scales the features to a distribution with mean of zero and variance of one.

In stage 2, regression models were constructed with structural and elastic properties as predictors and plastic properties as outcomes. Predictors with significant correlation with plastic properties were considered to this aim, while predictors with low correlation were excluded. The most basic form of regression is linear regression, in which least squares approximation is used on a set of explanatory variables to predict an outcome. Penalization of regression coefficients was also considered by means of Ridge regression with L2 regularization ^36^ and Lasso regression with L1 regularization ^37^. All models were trained and tested following 10–fold cross–validation. This means that data was divided into ten parts, where the first 10% is used for testing and the rest for training. The selection procedure is repeated ten times to ensure that each part is used for testing ^38^. In stage 3, two metrics were calculated to assess the performance of the regression models. The coefficient of determination ($R^2$) was calculated on both the training and testing set to evaluate the capability of predictors to explain the outcomes. $R^2$ is defined as

3 $$\begin{aligned} R^2 = 1 -\frac{\sum _{i=1}^n (y_i-\hat{y}_i)^2}{\sum _{i=1}^n (y_i - \overline{y})^2}, \end{aligned}$$

where *n* is the number of observations, $y_i$ is the true value, $\hat{y}_i$ is the predicted value by the regression model and $\overline{y}$ is the mean value of *y*. Since $R^2$ varies from zero (no predictive capability) to one (maximum predictive capability), its value can be represented as a percentage.

The root mean squared error (RMSE) was obtained to assess the prediction capability of the models on both the training and testing sets. RMSE is defined as

4 $$\begin{aligned} \hbox {RMSE} = \sqrt{\frac{1}{n} \sum _{i=1}^n (y_i- \hat{y}_i)^2}. \end{aligned}$$

Data analysis and ML modeling were carried out using Pandas ^39^ and Scikit–learn ^40^ libraries for the Python programming language.

## Results and discussion

### Mechanical behavior

**Figure 2:**
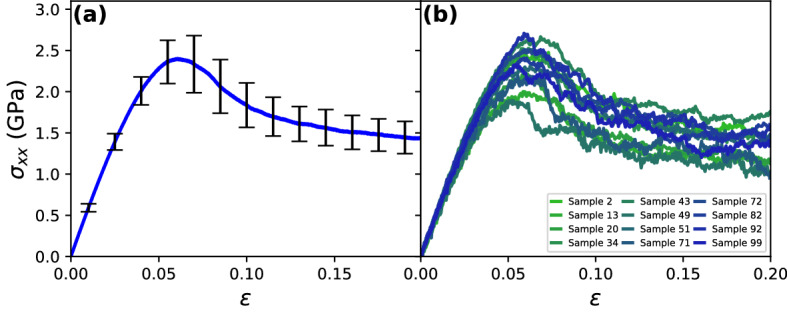
(**a**) Average stress–strain curves for the 100 samples. Standard deviations are denoted by the black bars. (**b**) Stress–strain curves of 12 samples were selected randomly.

Tensile tests were carried out to obtain mechanical properties from the stress–strain curves. Figure 2a shows the average curve calculated by considering all samples and the black bars denote standard deviations. Elastic behavior is distinguished up to $\sim 0.03$ strain. Here, the standard deviations are small, which is expected since elastic regime does not differ remarkably among different CuZr MG compositions ^41^. During the onset of plasticity, the standard deviations increase significantly, corresponding to different values of yield ($\sigma _Y$) and ultimate tensile stress ($\sigma _{UTS}$). Qualitatively, all samples display a similar softening behavior, but they differ quantitatively in the flow stress. Standard deviations do not vary significantly during this stage, indicating that the differences between curves remain constant with strain. In order to obtain more details of the elastic and plastic behaviors, the stress–strain curves of 12 samples randomly selected are shown in Fig. 2b. Overall, all curves exhibit the same pattern of the average curve, and the differences in stress are in the range of the standard deviations. The large ductility displayed by all samples has been previously reported in both experimental and simulation studies when dimensions are in the order of 100 nm. This behavior has been attributed to size effects that suppress deformation localization and failure ^42–44^. In addition, radial distribution functions were calculated to check whether the samples remained in amorphous state during the tests. As shown in Fig. S2 of the supplementary material, no relevant changes were found. Following our results, it can be concluded that all samples presented comparable elastic and plastic regimes. From a statistical point of view, this indicates that the possibility of observing outliers is reduced.

### Univariate analysis

An overview of the structural and mechanical properties, together with other variables such as temperature and atomic composition, is provided by means of univariate statistical analysis. The results are shown in Table 1. Cu present a mean of 50.38, with values ranging from 36 to 64 as expected from the initial configurations. The number of atoms ($N_a$) is relatively small for the current trends in MD simulations. However, such issue is overcome by considering a large number of cases, and thus, resulting in a wide range of MG properties. The length of the samples ($L_x$) is significantly larger than both the width and height ($L_y, L_z$) as expected from the construction procedure. All cooling rates ($R_c$) fall into the range of 1 to 10 $\times 10^{10}$ K/s, with most cases around $4-5 \times 10^{10}$ K/s. These values are widely adopted in MD studies due to limitations in simulation times ^32,33,45,46^. Regarding temperature, typical values of MD simulations were considered. with limiting cases at 50 and 290 K. Temperature is known to induce fluctuations at the atomic scale and to induce plasticity at lower yield stress ^46,47^. Thus, the range considered here leads to different stress values in the plastic regime. The coordination number (*CN*) shows little variation, ranging from 12.3 to 13.1, which can be explained from Voronoi polyhedra analysis. As discussed in previous works, large populations of high–centrosymmetric polyhedra are usually found in CuZr MGs, with coordination numbers close to 12. Slightly higher values are observed here, since the *CN* calculated from radial distribution functions consider the first neighbor shell with a cutoff radius of 3.7 Å, in contrast to the method of Voronoi polyhedra that considers the neighboring atoms in the Voronoi cell ^48^. These populations of high–centrosymmetric polyhedra lead to average degree of five–fold local symmetry (*W*) close to 0.57, similar to the findings of other authors ^49^. Reported values of Young’s modulus (*E*) and Poisson’s ratio ($\nu$) of CuZr MGs are close to 50–60 GPa and 0.3–0.4, respectively, with increasing values at larger contents of Cu. Such ranges are in agreement with results published in literature ^50–52^. The yield stress ($\sigma _Y$), ultimate tensile stress ($\sigma _{UTS}$), drop stress ($\sigma _D$), and flow stress ($\sigma _F$) have small standard deviations, which is expected from the relatively small black bars observed in the average stress–strain curve. Although our results are in the range of those reported in previous works ^24,26,52,53^, the comparison is not straightforward since boundary conditions can affect the stress values ^47^. Resilience ($u_R$) and toughness ($u_T$) also present small variations, with values close to 0.03 and 0.33, respectively, as observed from the mean and the median. Unfortunately, little information is available in the literature regarding both quantities for CuZr MGs, being difficult to deliver comparisons. The distribution of each property is shown in Sect. 3 of the supplementary material.

**Table 1:** Descriptive statistics for each feature under consideration.

Variable	$\overline{x}$	*s*	Min	Q1	Median	Q3	Max
Cu (%)	50.38	8.67	36.00	42.75	49.50	58.00	64.00
$N_a$	33291	13231	12880	22334	31460	40658	72000
$L_x$ (nm)	21.0	3.2	16.2	18.1	20.9	23.8	25.9
$L_y$ (nm)	8.4	1.0	6.5	7.5	8.4	9.4	9.7
$L_z$ (nm)	3.2	1.1	1.6	2.3	3.2	4.2	4.9
$R_c$ ($\times 10^{10}$ K/s)	4.97	3.59	1.00	1.75	4.00	8.00	10.00
*T* (K)	180.6	74.2	50.0	130.0	180.0	235.0	290.0
*CN*	12.7	0.2	12.3	12.5	12.7	12.9	13.1
*W*	0.57	0.027	0.54	0.55	0.56	0.58	0.64
*E* (GPa)	56.99	3.95	47.28	54.89	56.83	59.48	67.85
$\nu$	0.40	0.01	0.39	0.39	0.39	0.40	0.42
$\sigma _Y$ (GPa)	1.74	0.19	1.29	1.63	1.74	1.86	2.23
$u_R$ (GJ/m$^3$)	0.030	0.005	0.018	0.028	0.030	0.033	0.041
$\sigma _{UTS}$ (GPa)	2.51	0.29	1.89	2.30	2.49	2.72	3.31
$\sigma _{D}$ (GPa)	1.03	0.15	0.72	0.91	1.05	1.15	1.38
$\sigma _F$ (GPa)	1.48	0.19	1.10	1.33	1.48	1.59	2.09
$u_T$ (GJ/m$^3$)	0.332	0.040	0.251	0.309	0.328	0.358	0.443

### Correlation between variables

**Figure 3:**
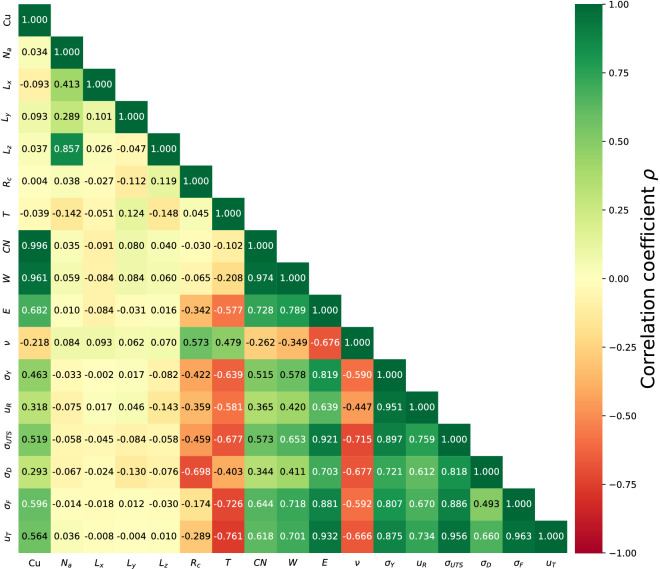
Spearman’s correlation heatmap for all variables. Red color represents negative correlations and green corresponds to positive correlations.

Possible correlations between the 17 different features were explored by means of Spearman’s correlation coefficient resulting in the heatmap shown in Fig. 3. Limiting values of -1.00/1.00 correspond to a perfect negative/positive monotonic association, while zero values represent no association. Correlations between features with themselves are excluded from the analysis. From the heatmap, three evident relationships can be distinguished. Each of them correspond to a strong correlation between $L_x$, $L_y$, $L_z$ with $N_a$, which is straightforward from the direct relationship between dimensions and total number of atoms.

Both *CN* and *W* present strong positive and negative correlations with Cu. It has been previously identified that Cu species are associated to high–centrosymmetric polyhedra ^24,25^, which in turn possess larger coordination numbers and increased degree of five–fold local symmetry ^49,54^. Although Zr atoms are also related to polyhedra with high *CN* and *W* values, such as $\langle 0, 1, 10, 4 \rangle$ and $\langle 0, 1, 10, 5 \rangle$, these populations are usually small compared to Cu species ^25,55,56^. From these considerations, it is straightforward to understand the high correlation between *CN* and *W*. $N_a$, $L_x$, $L_y$, $L_z$ exhibit no correlation with other variables, indicating that dimensions do not have any relevant effect on the materials properties. The cooling rate presents negative correlations with most elastic and plastic properties. This behavior is expected since $R_c$ has been identified as a rejuvenation mechanism that leads to increased energy states ^57^. A similar pattern is distinguished for temperature, where negative relationships between *T* and *E*, $\sigma _{UTS}$, $\sigma _D$, $\sigma _Y$ are observed as reported in previous works ^58,59^. This can be interpreted from kinetic energy, since it favors atomic mobility and nucleation of shear transformation zones (STZs). The positive correlation between *T* and $\nu$ has been previously reported in the literature for conventional alloys and CuZr MGs ^60,61^ and it can be understood from the enhanced lateral strain at higher temperatures. *CN* and *W* exhibit positive variations with elastic and plastic properties. Larger values of these quantities correspond to highly–densely–packed structures which are less prone to deform under external perturbation ^62^. Both elastic and plastic properties, excluding the Poisson’s ratio, show positive variations with Cu (and thus negative variations with Zr). Previous works have reported that high–centrosymmetric polyhedra compose the structural backbone of CuZr MGs. Hence, larger Cu contents endow enhanced rigidity, whereas the opposite behavior occurs with Zr atoms ^63^. In the case of Poisson’s ratio, increased stiffness reduces the degree of lateral strain, explaining the negative correlation between Cu and $\nu$. Such behavior has been reported for Cu contents below 60%, whereas a change of this trend occurs at larger contents ^64^. Finally, correlations between elastic and plastic properties are all positive as explained previously from stiffness and resistance. The exception here is $\nu$, whose value decreases as rigidity increases.

**Figure 4:**
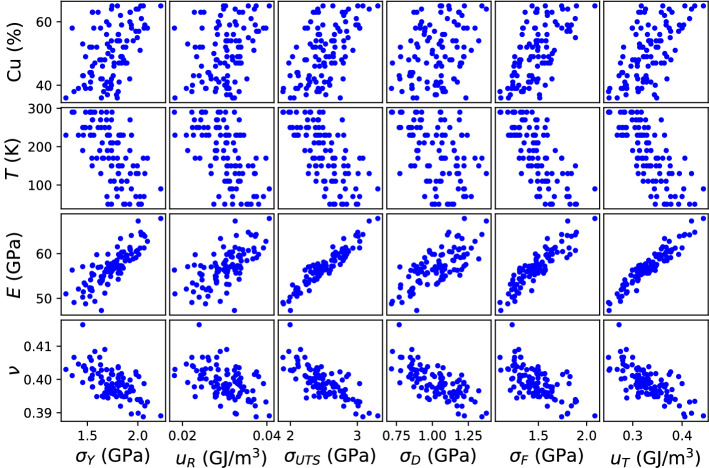
Relationships between materials properties and plastic properties in Cu$_{x}$Zr$_{100-x}$ MGs.

Among the structural and elastic properties, some of them presented high degree of correlation, particularly, Cu, CN, and W, which rendered values close to one. Since input features with large degrees of correlation can impact negatively the ML models, only Cu was retained and the others three were excluded. Materials properties significantly correlated with plastic properties ($\sigma _Y, u_R, \sigma _{UTS}, \sigma _D, \sigma _F, u_T$) were selected for further analysis. To this aim, the absolute value of each correlation coefficient was calculated and a threshold of 0.5 was set. Some features presented significant correlations with most plastic properties, but failed in some cases (see for instance Cu with $\sigma _Y$, $u_R$, and $\sigma _D$). In such situation, the threshold criterion is neglected and the feature is kept. To further understand the relationship between these features, scatter plots were constructed resulting in Fig. 4. Monotonic trends are clearly distinguished in all cases. Here, large degrees of dispersion are observed for Cu and *T* as reflected from a relatively low correlation coefficient in the range of 0.3–0.7 (see Fig. 3). For the elastic properties *E* and $\nu$, the degree of dispersion is reduced, which is explained from the higher values of $\rho$ in the range of 0.6–0.9. Since plastic properties are correlated with structural and elastic properties, a ML model can be constructed to predict the plastic behavior of MGs. In the following section, regression models are proposed to accomplish this task.

### Machine learning analysis

ML models were prepared using highly correlated features. The materials properties Cu, *T*, *E*, $\nu$, were employed as predictors (input features), and the six plastic properties $\sigma _Y$, $u_R$, $\sigma _{UTS}$, $\sigma _F$, $\sigma _D$, $u_T$ were set as outcomes, resulting in the following regression models

5 $$\begin{aligned} y_i= w_{i,0}+w_{i,1}\hbox {Cu}+w_{i,2}T+w_{i,3}E+w_{i,4}\nu , \end{aligned}$$

where $i=[1,6]$ correspond to $\{\sigma _Y, u_R, \sigma _{UTS}, \sigma _F, \sigma _D, u_T \}$, and $w_{i,j}$ are the regression coefficients. What is remarkable of this model is that it aims to predict the plastic behavior based on materials properties that do not involve irreversible deformation of the sample. Two different metrics were obtained to assess the performance, $R^2$ and RMSE.

**Figure 5:**
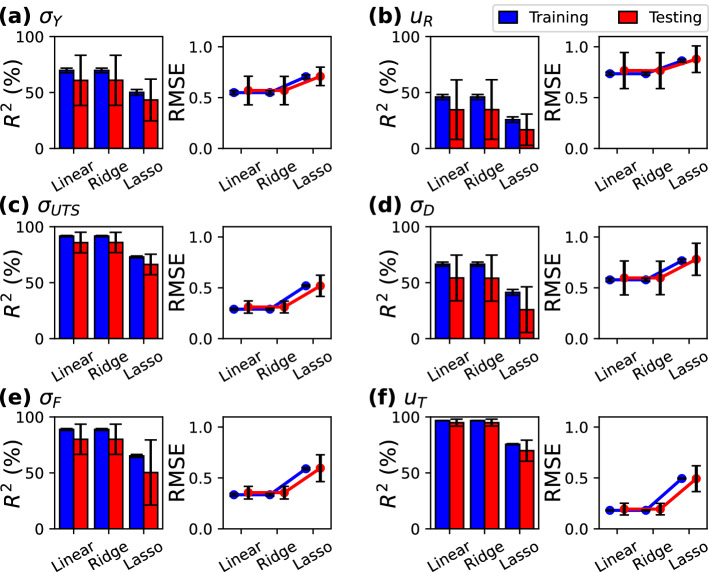
Coefficient of correlation ($R^2$) and RMSE for the plastic properties (**a**) $\sigma _Y$, (**b**) $u_R$, (**c**) $\sigma _{UTS}$, (**d**) $\sigma _D$, (**e**) $\sigma _F$, and (**f**) $u_T$. RMSE curves were horizontally–displaced for visualization purposes.

Figure 5 shows the results for linear, Ridge, and Lasso regressions. The predictive capability of both linear and Ridge regressions is quite similar. Remarkable values are observed for $R^2$ in the case of $\sigma _{UTS}$, $\sigma _F$, and $u_T$ ($>80\%$), whereas it decreases for $\sigma _Y$, $\sigma _D$ ($\sim 70\%$) and $u_R$ ($\sim 50\%$). Interestingly, Lasso regression rendered lower coefficients of determination for the six plastic properties, with values above $\sim$70% for two of them ($\sigma _{UTS}, u_T$) and below $\sim$60% for the other four. A similar behavior is distinguished for RMSE, where linear regression and Ridge regression delivered comparable results, whereas Lasso rendered higher values. Lasso algorithm is known to reduce the number of features during the regression process. Here, only two features were retained: temperature and Young’s modulus. Therefore, the lower performance of Lasso can be attributed to the exclusion of relevant materials properties for regression. Larger standard deviations were obtained for $\sigma _Y$, $u_R$, and $\sigma _D$ when compared to the other plastic properties. Extraction of both $\sigma _Y$ and $u_R$ from the stress–strain curve is not straightforward as compared to other properties, which leads to higher variability. In the case of $\sigma _D$, its calculation involves the difference of $\sigma _{UTS}$ and $\sigma _F$. Therefore, the standard deviation of $\sigma _D$ depends on the variability of the two aforementioned quantities. It is worth to mention that the training set rendered better performance on both metrics ($R^2$ and *RMSE*) and lower standard deviations than the testing set, since the ML models were constructed using the testing set. Overall, Linear and Ridge regressions exhibited the better performance for the prediction of plastic properties due to relatively high $R^2$ and low RMSE compared to the Lasso regression, suggesting that both regression models can be useful tools to determine the plastic behavior of MGs.

From the previous discussion, in the three models $\sigma _{UTS}$ and $u_T$ presented the largest degree as reflected from $R^2$. Some insights can be gathered from the relationship between both plastic properties with the predictors in Eq. (5). It has been well–established that Cu species are related to high–centrosymmetric structures in CuZr MGs, leading to increased resistance but lower ductility ^25,64^. Temperature is another parameter that strongly affects plastic behavior. As reported for both crystalline and amorphous metals, higher temperatures reduce the ultimate tensile stress and increase ductility due to enhanced atomic mobility. In contrast, lower temperatures hinder the onset of plasticity and reduces ductility ^65–67^. The Young’s modulus has been reported to be in direct relationship with the fracture strength ^68,69^, whereas for the Poisson’s ratio, higher values correspond to a higher possibility for the material to shear under external stress ^70–72^. Therefore, the four predictors are strongly related to plastic behavior of MGs, and when combined together, regression models can be constructed for the prediction of ultimate tensile stress and toughness.

## Conclusion

Research of metallic glasses at the atomic scale is an active field that has been evolving through the years. Relationship between plastic behavior with other materials properties has been observed, leading to the question whether plasticity can be systematically predicted from the initial structure. In this work, statistical analysis showed that strong correlation exists between certain properties. For example, Cu content, Young modulus, and temperature were correlated with five–fold local symmetry, yield stress, flow stress, among others. These observations encouraged the development of supervised learning for the prediction of plastic behavior. Thus, by using materials properties, such as species content, temperature, and elastic properties, regression models were constructed for the prediction of ultimate tensile stress, drop stress, flow stress, and toughness. Linear and Ridge regression accomplished this task with the better performance as observed from the high coefficient of determination for the prediction of three plastic properties, whereas Lasso regression presented overall lower prediction capability. An interesting result from the machine learning models is that plastic behavior can be predicted from properties whose calculation do not involve irreversible mechanical deformation of the sample. This formulation offers new opportunities for the prediction of mechanical behavior of materials without carrying out deformation tests.

With the advances in computational simulations, increasing data is available, favoring the use of supervised learning models. This work showed that prediction of materials properties benefits from these state of the art techniques. Since molecular dynamics simulations involve thousand to millions of atoms, more studies using machine learning methods should emerge in the near future. While our work was limited to CuZr MGs, we expect that upcoming works could extend this contribution to other amorphous solids.

## Supplementary Information

Supplementary Information.

## Data Availability

The datasets used and/or analysed during the current study available from the corresponding author on reasonable request.
