# Supplementary material for: Machine learning modeling for the prediction of plastic properties in metallic glasses
Source: Sci Rep. 2023 Jan 7;13:348. doi: 10.1038/s41598-023-27644-x (PMC9825623; doi:10.1038/s41598-023-27644-x)
Supplement: Supplementary file 1 — Supplementary Information. [file 41598_2023_27644_MOESM1_ESM.pdf]

Supplementary Material
Machine learning modeling for the prediction of plastic properties in metallic glasses

Nicolás Amigo[1], Simón Palominos[2], and Felipe J. Valencia[3,4]

[1]Facultad de Ingeniería, Arquitectura y Diseño, Universidad San Sebastián, Bellavista 7, Santiago, 8420524, Chile
[2] Escuela de Ingeniería Industrial, Facultad de Ciencias, Ingeniería y Tecnología, Universidad Mayor, Santiago, Chile
[3] Departamento de Computación e Industrias, Facultad de Ciencias de la Ingeniería, Universidad Católica del Maule, Talca 3480112, Chile.
[4] Centro para el Desarrollo de la Nanociencia y la Nanotecnología, CEDENNA, Avda. Ecuador 3493, Santiago 9170124, Chile

1 Definition of structural and mechanical properties

Structural properties were calculated according to the following definitions. Percentage of Cu species was obtained from the total atomic composition. The coordination number, CN, was obtained from the radial distribution function up to the first neighbor shell. The average degree of five–fold local symmetry, W, was calculated as

$$W = \sum_{i=1}^{n} P_i \times f_i^5, \tag{1}$$

where n is the total number of polyhedron types, P_i is the fraction of polyhedron type i and f_i^5 is the five–fold local symmetry of polyhedron type i defined as

$$f_i^5 = \frac{n_i^5}{n_i^3 + n_i^4 + n_i^5 + n_i^6}. \tag{2}$$

Here, n_i^j is the number of j–edged faces of polyhedron type i.

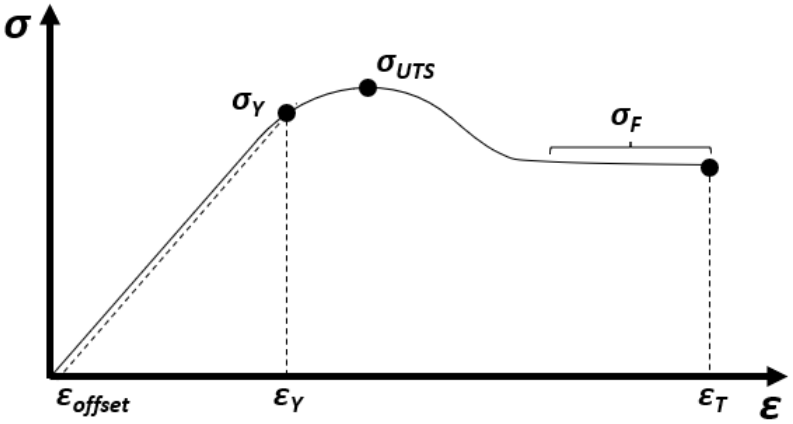

Figure S1: Scheme of plastic properties calculated from the stress–strain curve.

Mechanical properties were obtained from the stress–strain curve of each tensile test. A scheme of this curve is shown in Fig. S1. The Young Modulus, E, was obtained from the slope of the linear part of the curve. The yield stress, σ_Y, was calculated following the offset criterion with $\varepsilon_{offset} = 0.002$. The ultimate tensile stress, σ_{UTS}, was obtained from the maximum stress of the curve. The flow stress σ_F was defined as the average stress in the

range of 0.15–0.20 strain. The stress drop, σ_D, was obtained as the difference $\sigma_{UTS} - \sigma_F$. The Poisson's ratio was calculated as

$$\nu = -\frac{\varepsilon_{lateral}}{\varepsilon_{longitudinal}}. \tag{3}$$

The resilience and the toughness were calculated as

$$u_R = \int_0^{\varepsilon_Y} \sigma d\varepsilon, \tag{4}$$

$$u_T = \int_0^{\varepsilon_T} \sigma d\varepsilon. \tag{5}$$

2 Radial distribution functions

In order to check whether the samples remained in amorphous state during the tensile tests, radial distribution functions were calculated for two cases, sample 13 and sample 76. The former corresponds to a $Cu_{36}Zr_{64}$ MG and the latter to a $Cu_{64}Zr_{36}$ MG. The radial distribution functions for three different strains are shown in Fig. S2. In both cases, the functions do not show significant variations, except for a minor reduction in intensity of the first peak (first neighbor shell). Thus, in general terms, the samples keep their initial amorphous structure during the tensile tests. The same behavior was observed for the other samples (not shown here).

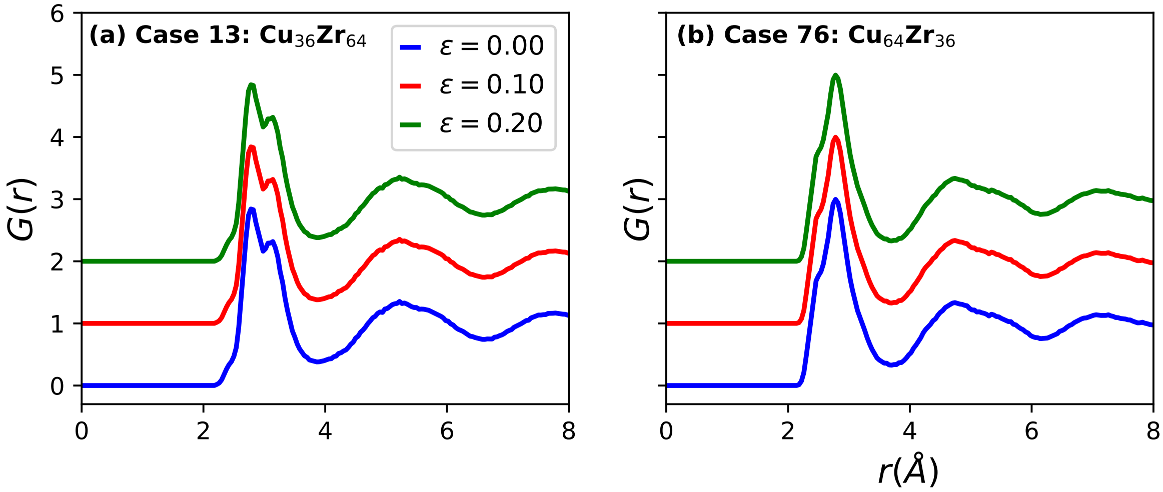

Figure S2: Radial distribution functions for two samples at three different strains.

3 Distributions of properties

The distribution of each property is shown in the following. N_a, L_x, L_y, L_z, T, were obtained from a uniform distribution as provided by the random number generator in the Python programming language. R_c was partially obtained from a uniform distribution, since the values 3×10^{10}, 6×10^{10}, 7×10^{10}, 9×10^{10} were excluded to avoid complications when setting the simulation time for the quenching stage. Otherwise, awkward numbers of simulation time would have been required to achieve the exact cooling rate. CN, W, E, u_R, ν, σ_Y, σ_{UTS}, σ_F, σ_D, u_T were calculated from the tensile tests. Calculation of elastic and plastic properties reveal that their values converge to normal distributions, indicating that such properties do not vary significantly from sample to sample.

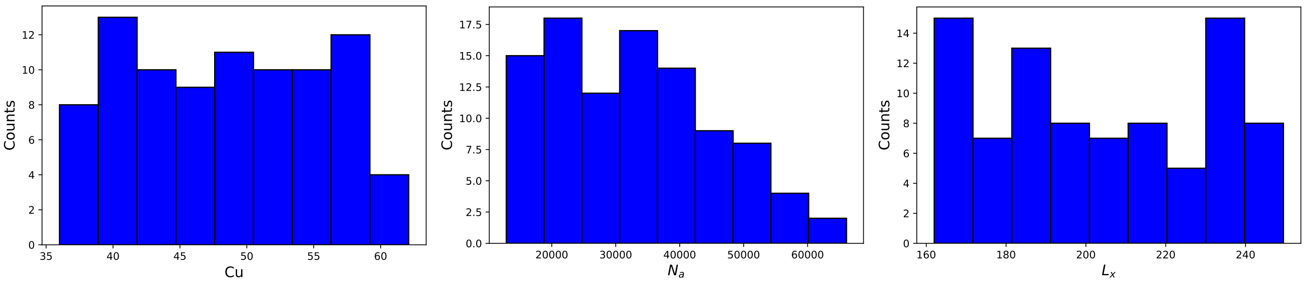

Figure S3: Distribution of (a) Cu, (b) N_a, and (c) L_x.

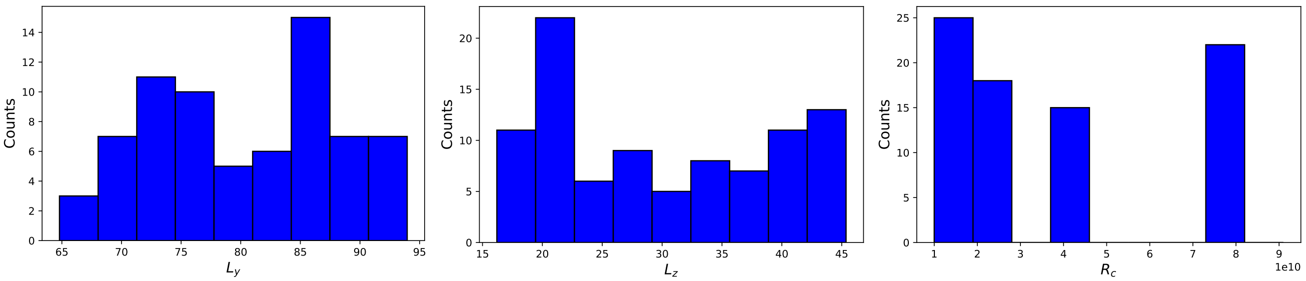

Figure S4: Distribution of (a) L_y, (b) L_z, and (c) R_c.

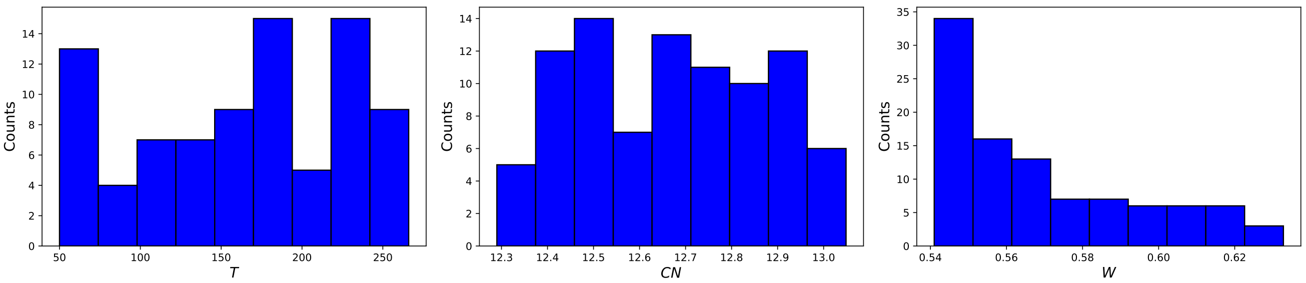

Figure S5: Distribution of (a) T, (b) CN, and (c) W.

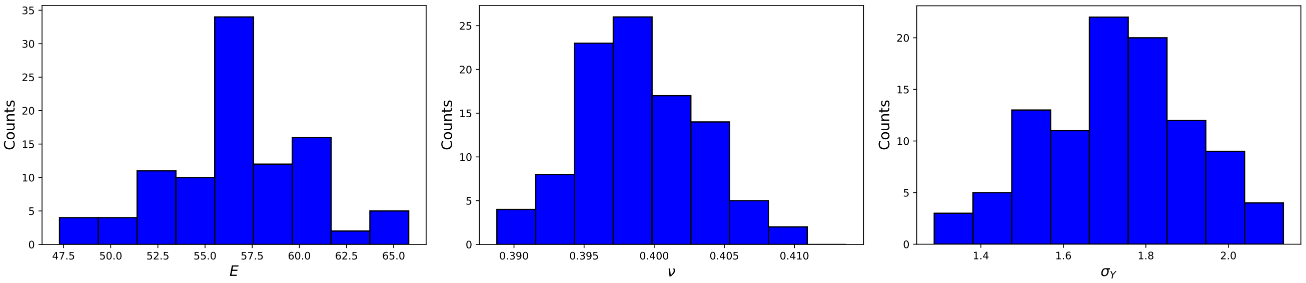

Figure S6: Distribution of (a) E, (b) ν, and (c) σ_Y.

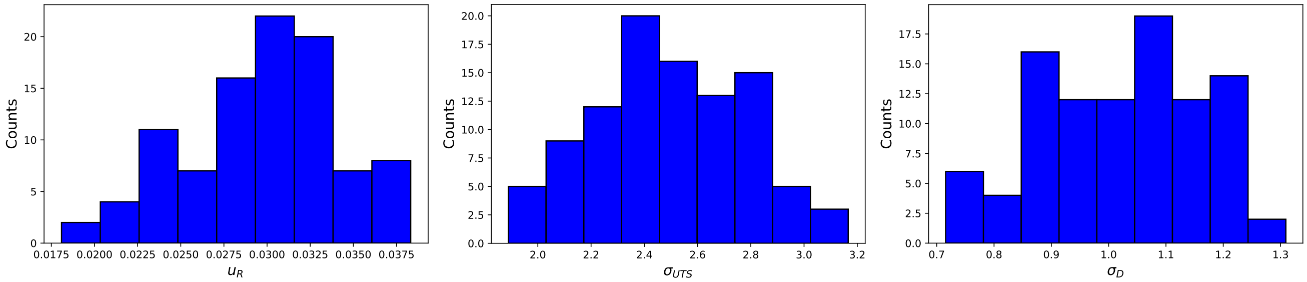

Figure S7: Distribution of (a) u_R, (b) σ_{UTS}, and (c) σ_D.

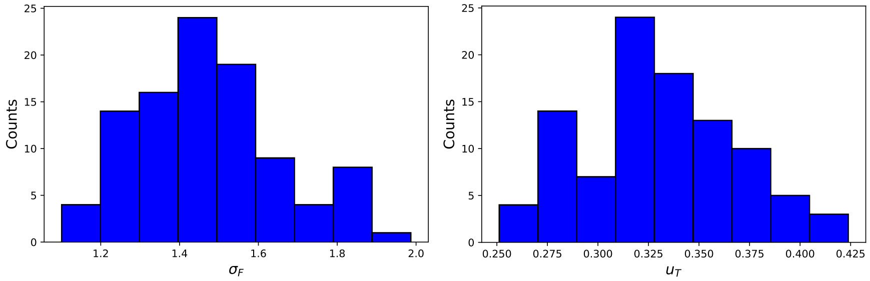

Figure S8: Distribution of (a) σ_F, and (b) u_T.
